# The Birth of *JBMR Plus*

**DOI:** 10.1002/jbm4.10004

**Published:** 2017-05-09

On behalf of the ASBMR and our publishing partner Wiley, I am proud to announce the birth of *JBMR Plus*, a new online‐only Open Access journal.

The field of bone and musculoskeletal research continues to grow and evolve as research moves into the promising world of translational and regenerative medicine. Importantly, the nexus between an increased understanding of the molecular signaling systems within and between bone cells, led by bone biology research, and careful clinical investigation has led to the development of novel and targeted therapeutic agents to treat osteoporosis and prevent fragility fractures.

Bone and mineral research has grown exponentially since the late 1980s, and its development has been allowed by excellence in the fields of mouse genetics, basic bone cell biology, preclinical research in animal models, and clinical research involving large multicenter, randomized controlled trials. In parallel, bone imaging techniques for measuring skeletal fragility in animal models and humans have also rapidly evolved, so that new methods for measuring trabecular and cortical microstructure with high resolution have been discovered. Significant basic science discoveries allowing translation into important clinical applications are now being made at astonishing speed.

At the same time, there is a growing trend toward making scientific research open access. This trend is being fostered both by institutional and funder mandates to publish in this format and by the desire for equity of access allowing investigators to publish their work so it is immediately available to all. As a fully open‐access journal, all content in *JBMR Plus* will be free to download, read, and share.

Synchronously, there is also widespread concern about the surprising lack of reproducibility in preclinical research studies. The leaders of the National Institutes of Health in the United States, Francis S Collins and Lawrence A Tabak, have addressed this concern by announcing a number of actions, including supporting researcher training on enhanced reproducibility, increasing the transparency of research findings, and enforcing good experimental design. In this regard, the ARRIVE guidelines for reporting animal research are designed to improve bioscience reporting. These needs for reproducibility and excellence in study design, as well as those for open access, will be well served by the creation of *JBMR Plus*.

There is also growth in interdisciplinary research involving endocrinology, geriatrics, orthopedics, and rheumatology. These factors provide a timely opportunity to develop *JBMR Plus*, which is designed to serve all members of this multidisciplinary field by publishing urgent research of the highest quality and impact. *JBMR Plus* will publish original research, reviews, detailed protocols and methods, and special articles in basic, translational, and clinical science relevant to bone, musculoskeletal metabolism, and regenerative medicine research. We welcome research on osteoimmunology, as well as research on fat, muscle, cartilage, and kidney interactions with bone. Clinical studies, including trials and observational studies, and epidemiology and pharmacoepidemiology studies are encouraged. *JBMR Plus* also welcomes manuscripts on orthopedics, stem cell therapies, specialized biomechanics protocols, novel imaging techniques, and big data.

The first three articles accepted into *JBMR Plus* highlight the scope of our journal and that we are on track to achieve our goals. In the first, Gorvin and colleagues^1^ established a mutant mouse model that is haplosufficient for adaptor protein‐2 sigma subunit (AP2σ), which forms a heterotetrameric complex with AP2α, AP2β, and AP2 μ subunits that is pivotal for clathrin‐mediated endocytosis. AP2σ loss‐of‐function mutations impair internalization of the calcium‐sensing receptor (CaSR), a G‐protein–coupled receptor, and cause familial hypocalciuric hypercalcemia type‐3 (FHH3). Interestingly, homozygous *Ap2s1^del17/del17^* mice had embryonic lethality, indicating an important role for AP2σ in embryonic patterning and organogenesis.

In the second, investigating factors stimulating osteoblast differentiation from mesenchymal stromal cells, Brum and colleagues^2^ demonstrate in a series of elegant experiments that *CLIC3* (chloride intracellular channel protein 3) is a novel gene regulating osteoblastic differentiation and enhancing bone formation. It promotes these effects through its interaction with NIMA‐related kinase 9 (NEK9) and phosphatidylserine synthase 1 (PTDSS1). The specificity of *CLIC3* to promote the osteoblastic lineage and osteoblast differentiation could make it a potential target for future bone analogic drugs. In the third, Lee and colleagues^3^ report the first case of fibrous dysplasia of bone associated with avascular necrosis of the femoral head without an antecedent fracture.

As Editor‐in‐Chief, one of my aspirations for *JBMR Plus* is that it helps to accelerate the research into bone biology that has underpinned the recent innovative therapeutic advances in bone and mineral research. In this regard, two highly competent and outstanding researchers, Drs Teresita Bellido and Bo Abrahamsen, will support me as Deputy Editors in the initial phase of the *JBMR Plus*'s development. As a sister journal to *JBMR*, we will also be able to access a remarkable pool of experienced, expert reviewers who have made *JBMR* the most highly regarded international journal in bone and mineral research. Such support allows us to make *JBMR Plus* a rapidly responsive and innovative home for new discoveries that will ultimately improve global musculoskeletal health.

Finally, to quote from the esteemed Dr Larry Raisz, the inaugural Editor‐in Chief of *JBMR*'s first editorial, “Our success will depend on the continued willingness of the members of the ASBMR and other workers in bone and mineral research throughout the world to submit their best work to us. To achieve this, we need to provide rapid reviews of high quality, make fair decisions, and help our authors to publish their best work promptly and in the best possible form.” At *JBMR Plus*, we now aim to honor Larry's memory by achieving these goals to make his “grandchild” a great success!


Peter R Ebeling, AO, MBBS, MD, FRACP
Editor‐in‐Chief
